# Understanding public perspectives on direct-to-consumer pharmacogenomic testing in the UK: a qualitative study

**DOI:** 10.1038/s41397-026-00425-1

**Published:** 2026-07-25

**Authors:** Amy Mathieson, Lisa Brunton, Stephanie Gillibrand, Ramona Moldovan, John H. McDermott

**Affiliations:** 1 Centre for Primary Care and Health Services Research, University of Manchester, Greater Manchester, England UK; 2 Division of Evolution & Genomic Sciences, University of Manchester, Greater Manchester, England UK; 3 Manchester Centre for Genomic Medicine, Saint Mary’s Hospital, Manchester, Greater Manchester, England UK; 4 Department of Psychology, Babeș-Bolyai University, Cluj-Napoca, Romania

**Keywords:** Genetic testing, Health services

## Abstract

Direct-to-consumer (DTC) pharmacogenomic (PGx) testing is expanding rapidly in the UK, yet no dedicated regulatory framework currently governs these services. Although a 2021 parliamentary inquiry recommended stronger safeguards and clearer technical standards for genomic testing, these proposals have not been applied to PGx. This study explores public attitudes toward DTC PGx testing, focusing on expectations for pre-test information, quality standards, and NHS data use. We conducted focus groups with members of the public, both with and without prior experience of purchasing DTC PGx tests, or other online health tests. Focus groups were audio-recorded with consent, transcribed, and analysed thematically. We identified three themes: (mis)understanding towards and awareness of DTC PGx testing; altruistic motivation and equity concerns; and (mis)trust. Participants were generally enthusiastic about PGx testing, as long as issues of equity, data protection, and regulation were addressed, with data sharing concerns being particularly prominent.

## Introduction

In recent years, there has been a rapid growth in the availability of direct-to-consumer (DTC) pharmacogenomic (PGx) testing, with a growing number of companies offering this service to members of the public in the United Kingdom (UK). A range of general regulations currently apply to genomic tests marketed to UK consumers [1–7]. However, the UK does not currently have a specific legal or regulatory framework in place to govern DTC genomic testing or the companies who provide these services.

The potential costs and benefits of introducing regulation for DTC genomic testing were examined by the UK Parliament’s House of Commons Science and Technology Committee and published as a report in 2021 [8]. The inquiry received written submissions from a wide range of stakeholders including clinical professional organisations, academics, charities, industry partners and industry bodies. Many submissions proposed updating DTC genomic test regulations to raise standards, protect consumers and the NHS, and increase public trust in DTC genomic tests [8]. The report made several recommendations, including strengthening pre-test safeguards, through improved consumer information or access to genetic counselling. It also called for improved quality assurance through the development of clear technical standards, intended to ensure that tests could be used and trusted by the NHS, and for extending the scope of DTC genomic tests’ performance requirements to cover clinical as well as analytical performance [8].

More recently, in 2025, The Royal College of General Practitioners, in collaboration with partners and stakeholders, issued guidance on DTC genomic testing for healthcare professionals working in primary care and community settings [9]. The guidance advises healthcare professionals not to accept records from non-accredited laboratories or third-party interpretation services. It also recommends that patients who present DTC genomic test results should receive the same NHS care as those who have not undergone such testing, and that result should not affect access to treatment(s) that are not commissioned for that condition within the NHS.

However, to date, these recommendations have neither been adopted nor explored in the context of pharmacogenomic testing, nor have they been examined with patient and public representatives. We therefore undertook a study which aimed to explore public understanding and attitude towards DTC PGx testing, with a focus on attitudes and knowledge towards pre-test information, quality standards, and data access and reuse of data in the NHS.

This qualitative study forms part of a programme of work being undertaken by the Centre of Excellence in Regulatory Science in Pharmacogenomics, a collaboration between academia, industry, the NHS and Regulators aimed at accelerating the safe and effective implementation of PGx in healthcare [10].

## Methods

We report our methods using the Consolidated Criteria for Reporting Qualitative Studies (COREQ) checklist for qualitative research [11] (Supplementary File 1).

### Research team

Three researchers with experience in qualitative methodology facilitated the focus groups. All three interviewers were women, with no prior professional or personal relationship with any of the study participants.

### Study design

We conducted a qualitative study using online focus groups with members of the public to explore their understanding of, and attitudes toward, DTC PGx testing.

A purposive sampling strategy was used to recruit participants from three groups:

- Members of the public with experience purchasing DTC PGx tests
- Members of the public with experience purchasing other DTC healthcare tests online, but no PGx tests
- Members of the public with no experience purchasing any form of DTC healthcare testing.

Eligible participants were adults aged 18 years or older who were able to speak and read English and provide informed consent.

Ethical approval for this study was obtained from the University of Manchester Research Ethics Committee (UREC Ref: 2025-24099-43204).

### Recruitment process

Participants with experience purchasing DTC PGx tests were recruited through two genetic testing companies, which advertised the study to their client base. Clients who had opted into direct mailing were sent an email invitation containing the study email address and a link to a Qualtrics consent-to-contact form. Interested individuals either completed the form or contacted the research team directly.

Participants with experience purchasing other DTC healthcare tests, as well as those with no DTC testing experience, were recruited via the Research for the Future^1^ database. An email describing the study, including contact details and a link to the consent-to-contact form, was distributed through their mailing list.

In total, 88 individuals expressed an initial interest in taking part. All potential participants were sent a participant information sheet and given at least 24 h to consider participation before providing written informed consent via Qualtrics. Participants were also asked to complete a socio-demographic questionnaire via the Qualtrics platform ahead of the focus group.

We planned to conduct up to seven focus groups: two with participants who had purchased DTC PGx tests, two with those who had purchased other DTC healthcare tests, and up to three with those with no DTC testing experience. We purposively sampled to ensure a variation in sample, including different age groups, genders and ethnic groups. Table 1 provides a summary of the participants’ characteristics.

**Table 1:** Participants’ characteristics.

Characteristic	Participants n = 27
**Exposure to PGx (focus group number)**	
No experience of purchasing DTC online healthcare tests (focus groups: 1,2,5)	18
Experience of purchasing DTC online healthcare tests (focus groups: 3&6)	4
Experience of purchasing direct-to-consumer PGx (focus groups: 4&6)	5
**Gender**	
Female	18
Male	8
Non-binary	1
**Age range**	
Under 30	7
30–49	9
50–59	4
60–69	3
70+	4
**LTC/disability**	
Y	14
N	13
**Ethnic group:**	
White British	15
White other	4
Asian/Asian British – Indian	3
Asian/Asian British – Pakistani	1
Black/Black British – Caribbean/African	3
Arabic	1
**Index of Multiple Deprivation**	
Most deprived (deciles 1–3)	5
Medium deprivation (deciles 4–7)	10
Least deprived (deciles 8–10)	7
Missing	5

### Data collection

We conducted six focus groups with a total of 27 participants (between two and six participants per focus group – see Table 2). Data collection concluded after six focus groups as data saturation had been reached and no new concepts emerged. This indicated that the themes generated adequately reflected participants’ experiences and perspectives.

**Table 2:** Focus group and participant summary.

Focus group	Participant Group	Participants
FG1	No experience of purchasing any DTC online healthcare tests	P1, P2, P3, P4, P5, P6
FG2	No experience of purchasing any DTC online healthcare tests	P7, P8, P9, P10, P11, P12
FG3	Experience of purchasing online healthcare tests	P13, P14, P15
FG4	Experience of purchasing DTC PGx tests	P16, P17, P18, P19
FG5	No experience of purchasing any DTC online healthcare tests	P20, P21, P22, P23, P24, P25
FG6	Experience of purchasing online healthcare tests and/or DTC PGx tests	P26, P27

All focus groups were held online via Microsoft Teams. Focus groups were facilitated by LB, AM or SG, and co-facilitated by JM – a Clinical Genetics Speciality Registrar – or RM – a Genomic Counsellor. Topic guides, tailored for each participant group, were developed and subsequently reviewed by a Patient and Public Involvement and Engagement (PPIE) group, to ensure that the questions were relevant and understandable from a participant’s perspective. Following discussions with the PPIE group, minor changes were made to the topic guides, and the wording in the public-facing study material. Focus group discussions explored participants’ experiences or awareness of, and perspectives on, DTC PGx testing; their views on data access, sharing, and reuse by the NHS; and their perspectives on regulation and oversight of PGx testing, including pre-test information (See Box 1 for a summary of the topic guide questions).

Focus groups lasted between 46 and 63 min; the average length was 53 min. Focus groups were audio-recorded, transcribed, and fully anonymised to ensure confidentiality and compliance with GDPR. Field notes were made during, and shortly after, the focus groups. Data collection took place in October and November 2025. Participants received a £30 shopping voucher as a token of appreciation for their time.

Box 1 Summary of topic guide questions per participant group **Focus groups with people with no experience of purchasing DTC health tests and/or people with experience of purchasing other health tests**:

- Can you tell us what you know about private, paid for Pharmacogenomic testing?
- What do you think are the main concerns/benefits to participating in private, paid for pharmacogenomic testing?
- What would you like to see as a result of purchasing a Pharmacogenomic testing?

**Focus groups with people with experience purchasing DTC Pharmacogenomic tests:**

- Can you share your relevant experiences related to private, paid for, Pharmacogenomic testing?
- What motivated you to purchase a Pharmacogenomic test?
- What were your expectations? Were your expectations met?
- What did you understand about the purpose of the test?
- What type of information did you get before purchasing a test?
- Did you understand who would have access to your test results/report and how they would be used?
- Did you share your test results/report with your GP or healthcare professional? Why/why not?

**All focus groups:**

- What type of information would you want before ordering a DTC Pharmacogenomic test? How would you want to receive the information?
- How much trust do you have in the accuracy and reliability of the test results? What information would increase your confidence Pharmacogenomic tests?
- What checks and controls do you think are important for the use of these tests?
- How would you feel about your test results/report being shared with the NHS to be used in your direct care?
- How would you feel about your anonymised test results/report being shared with others (including NHS) to be used for research purposes?
- Would you support national policies that encouraged the sharing of DTC Pharmacogenomic data for NHS use?
- Do you have concerns about privacy or misuse of your genetic data/test results?

### Data analysis

Focus group data were analysed using thematic analysis, guided by Gale et al.’s modified Framework Approach. This approach has seven steps: (1) transcription, (2) familiarisation (3) identifying codes, (4) developing an initial coding framework, (5) applying the framework, (6) charting (summarising), and (7) interpreting the data [12]. Analysis was conducted using NVivo 15 qualitative data analysis software [13].

To develop the initial coding framework, LB and AM independently read and re-read three transcripts each, then met with SG to refine and agree on a preliminary set of codes. Coding responsibilities were shared across the research team. Once coding was completed, a single analyst (LB) reviewed the coded data, combined codes into themes, and mapped each theme. The full research team subsequently met to review and refine the themes and mapping.

## Results

Below we present the key themes analysed from the focus groups: (mis)understanding towards and awareness of DTC PGx testing; altruistic motivation and equity concerns; and (mis)trust. We present key quotes related to each theme in Tables 3, 4 and 5.

**Table 3:** Theme 1 supporting quotes.

**Lack of knowledge about DTC PGx testing**
*I don’t think I’d heard of it because this is different to, like, if there’s, like, breast cancer in your family, you can be tested to see if you carry the gene. Is it the same or…? The same idea, I guess, but this is purely how you react to medicines? Is that right? […]and I don’t think I’d heard of this with regards to the medicines specifically. I know I’ve been to the dentist before and got given a drug that made me feel sick and was told that it was common, but there was no talk of genetically that’s common, I guess. P2, FG 1*. *… people sometimes go down these testing routes, not necessarily knowing all about it, my understanding is that, currently, if you had genetic testing through the NHS, you would receive counselling beforehand, and after. […] Because it might be my decision to find out whether there is some life-threatening genetic condition that I have, but my children might not have made that same decision. And the other thing is, if it is, if it goes onto a patient’s record, so their medical record, when for example, GPs are asked to sign travel insurance, health insurance, annuity insurance… obviously with your permission, but you know, there’s another whole ball game there… P7, FG 2*. *No, I had no idea. I thought…my assumption was you’d have a test, it said you might have cancer or you might be liable to diabetes or you might be liable to different things, so I thought, well, what’s the point because unless it’s something I can do something about… But having listened to what John was saying, I’m thinking oh right, I’m taking statins, I’m quite happy with my statins as far as I know, but if I were not to be and I could have a test to see well, perhaps you’re not taking the right ones, there’s a better one. So in that situation I think I would, and I would pay for it if I thought that the GP would be able to do something about it. Do you see what I mean? P15, FG 3*. *I wasn’t aware that you could pay for genetic testing and that it could be done privately, that’s a new concept to me. However, I am aware of it on the NHS through the BRCA gene testing with …breast cancer. P25, focus group 5*.
**Limited awareness amongst healthcare professionals**
*“Yes. I guess the other thing I’d say is similar to what P1 was saying around…especially if the information is coming from outside of the NHS, I guess it’s making sure that NHS staff are educated correctly on… This is a perception but I imagine there could be, if they’re not correctly informed, some hesitation on going, oh, I don’t know if I actually trust the data anyway…So just making sure, especially when that’s outside of the NHS, I guess, that the NHS is able to make the best use of it and that internal training is there*.” P2, FG 1. *So, I think the test is quite good, but to be honest, when I showed, because at first I did, I had the diagnosis that I did privately, and then when I got transferred to NHS, my psychiatrist was like, oh sorry – that was his response, from NHS, yeah – sorry, but we don’t consider this, like, pharmacogenomic testing to be really, like, accurate. So, even if this drug is saying, like, you are a poor metaboliser, I’m still going to give you that one, just to see. And that is when I was like, okay, so what’s the point, like, I know like, that should give you some insight of how even NHS professionals think of the testing. Because I gave the documents, everything, like, he has history of my medicines, and you know, like, still like, ignored it, let’s say, and just did whatever he wanted. So that’s my experience. But I think, it’s future, I think it’s good, personally I’m for it. But I don’t think it’s still acceptable by everyone, by NHS as well, so that’s my opinion. P16, FG 4*. *“And so I think it’s probably important that GPs are already familiar with the testing and that they have…they’re familiar with it and they have confidence in it, otherwise, if you’re just presenting it to a GP, they could be quite dismissive of it, I think.”* P23, FG 5. *“I was just going to say the point about taking it to your GP. I don’t know if they would even understand it. I mean, this is pretty new stuff and their education or comprehension might be out of date and it might just be a waste of time*.” P20, FG 5.
***Need for greater public awareness and engagement***
*…communicating about pharmacogenomics is important people often, including myself, get confused exactly what you’re talking about, so having a clear narrative about a topic to avoid misconceptions is absolutely fundamental.” P1, FG 1*. *What comes to mind is, first and foremost, the need for public engagement. Because you see, the questions I have in mind are, what does it really mean for genetic testing to be done on me, and exactly what do you mean when you say it’s being done, or you are taking a sample for that. P8, FG 2*.

**Table 4:** Theme 2 supporting quotes.

**Personal or familial benefits of PGx**
*It would be like going to a tailor to get an outfit that would be a perfect fit for that person, rather than getting it off the hook which won’t look as good’ P9, FG 2*. You *can see an actual tangible benefit. […] An example of this is my eldest daughter went through about six or seven contraceptive pills before she found one that suited her […] something like that I can see why a specific medicine for a specific thing would be valuable.” P15, FG 3*. *I was given the full dose of statins, and he wanted to see how my body would process that going forward. So, after being on them for three months, I did the test, and very grateful that I did it. Because I was not processing that dose well, it was too strong for me. So, in conjunction with my cardiologist, they have halved the dose. Whereas, I would have sailed on for the rest of my life…taking the highest possible dose. With, obviously, the consequences that that can bring. So, I was really grateful. P18, FG 4*. *I think the pros are amazing. I mean, I think it’s just so beneficial and it’s exciting, isn’t it? So it’s, kind of, insightful. And if people can have a better quality of life on medication and have a really good response P24, FG 5, no experience*. *on the thoughts of personalising medicine, it seems that if, let’s say, if a particular size of dress wouldn’t fit for everyone, so why can’t the same be applicable in the medical practice whereby it should not be generalised methods for treatment, for drug administration, because even in our clothing or in our normal activities, there are things that are specific to individuals, so my size of dress might not be fitting for someone of my same age, sex and probably similar physique. P26, FG 6*.
**Wider societal benefits**
*It would save money of course in the long run, wouldn’t it, because masses amount of money goes on prescriptions and if you can guide them to the correct prescription in the first place there wouldn’t be thousands and thousands of tablets in other people’s houses just sitting there. P14, FG 3*. *So, the ability to not have to give people drugs that don’t work, and therefore save those costs, I think for me is the thing that stands out. It’s just whether the cost of actually doing the genetic testing and getting the people in to presumably blood test, to run those, whether that cost actually supersedes the cost that you’re saving would be the concern related to that for me. P6, FG 1*. *I think for me, my concerns would be, if the data is anonymised, it is specific for the purpose that it was taken for, then I have been asked to sign a consent form, and it’s with a credible organisation like NHS, I would be willing to have it shared, or used to make the world a better place. Because, and probably specific, or also for specific groups of people, like I’m from the Black community, I know not too much research has been done. So, I would be very willing to participate. But then, I have to be sure of all that, that it’s not being used for other purposes. P8, FG 2*.
***Potential for inequity of access and utilisation***
…*there are people that can’t afford to pay for it and that might be what rubs me the wrong way as well. If there’s a cost, obviously there’s such a wide spectrum of what people can pay for it, and when it feels like something that’s really fundamental, it feels like there should be tools, taking everyone on an equal playing field. P2, FG 1*. *Okay. I just wanted to say that this is, personalised medicine is the right step forward, but my issue is for the general public, for those who would not have money to pay for this kind of testing, what would they do? … for those that cannot afford it outside of the NHS, privately, what would they do then? P12, FG 2*. *I was just thinking, it’s probably not in the scope of your discussion group, but the moral issue of I can get mine done because I can afford it but my next door neighbour can’t because they can’t afford it, and I think that does give me anxiety. My test that I do, anyone can get it done, but I just have it done more frequently. I’m not saying it would stop me doing it, but it does give me concern about the fact that person A can do it, person B can’t. P15, FG 3*. *But the slight issue with it, it’s only going to be people who can afford the test at the moment. So, the demographic who’s going to be talking about that is quite limited. P19, FG 4*. *And also thinking about inclusivity, as in financial, because it’s available to pay private, not everyone’s going to be able to afford that, so is it going to create potential inequalities in health? P24, FG 5*. *But my only concern now is the cost for individuals because normally genomic sequencing is not cheap, even for a facility, so if an individual would be going for such…I don’t think…not everyone, the affordability would be a concern, an issue for me, yes. P26, FG 6*.

**Table 5:** Theme 3 supporting quotes.

**Culturally situated mistrust**
*What are the real dangers to me, are there any, even? I mean, I think that’s the thing we need to understand as the public. And probably for us to understand, what are the real benefits for me? Because when I am able to understand that, then some of those myths and fears could be dispersed, because as someone here said, especially for us Africans, who have a lot of myths, and fears, and religious feelings. But I’m asking myself, so you have my data, there’s nothing you can come and implant in me because you have my data, because I’ll not allow you to come and do it on my body. But you have my data, yes, it’s like when you have any other data. Maybe that’s a clarification most of us would need, for us to get to a level of comfort.”* P8, FG 2. *“Okay, where I come from, we value culture, and religious views. And when you do genetic testing, like, when you hear about it, or when they talk about it, people come with, people have fears, like they are scared about the results, and their suspicions set in. So, my question is, how will you bring people’s culture, and beliefs into consideration, how are you going to support individuals, emotionally, when they get results that are not palatable, how are they going to be supported psychologically?”* P12, FG 2. *But I think that clarification is so crucial to taking something like this forward. Because, you know, as P10 was saying, as soon as anyone mentions DNA testing, genetic testing, you immediately think about, almost like being, you know, everything about you completely undressed and bared to everyone, or anyone that wants to see it, and the implications of that*. P7, FG 2. *If it was renamed into like, drug interaction validity test, or like, something that just didn’t have the word genetics in it at all, it might sound like, at least it doesn’t have that instant alarm bells going off in someone’s head. So just renaming it, like, pharmacogenetics, I know it’s, or pharmacogenomics, I know that’s what it is, but it still has the word gene in it. So even if it is just something like, looking for side-effects, like, if it’s just so specialised to just the pharmaco interactions, and no one even has to think about genetics. Like, yeah, you could explain, like obviously, all the details, but if it’s just the initial alarm bells are taken off, it might make people more receptive to then hearing more about the actual science behind what they’re signing up for as well*. P11, FG2, no experience.
**Need for regulation and safeguards**
*And obviously, there’s lots of concerns regarding the data side of things, and how it would be used. I think there’s a trust issue within the general public of companies, et cetera. So, I think that would be a long process to go through for the general public*. P9, FG 2, no experience. *I think that most people in the UK have this understanding, maybe wrongly, maybe rightly, but it’s implied security with anything through the NHS. You know, we have NHS apps, we have records on there of GP appointments, blood tests, and everything else. But if this is going through private companies […] how can it be shown to be legitimate and ethical. Because we come back, as P9 just said, into the public general perception these days that, well if we don’t know about it, it’s not right, you know, and there’s bound to be something, and the data is bound to be stolen, and then I’m leaving this app*. P10, FG 2, no experience *I just want to echo [P19]’s sentiment here, that I agree fully. That my primary concern with this data is bad actors, and the primary bad actor in this sense is insurance companies, yeah. I have health insurance, and you know, that’s great and all. But they’re, I’m fully aware that they’re in it for themselves, right, they’re not…and that will just always be the case. And I am aware that if they could get their hands on genetic data that is predictive of disease, they will. And I don’t trust the Government either, necessarily, to handle data correctly, I don’t really trust them to handle non-medical data, and I certainly don’t really trust them to handle medical data in a very safe manner*. P17, FG 4.
**Need for regulation and safeguards**
*I would want to see really robust statutory safeguards around this before it becomes like a wild west and then we’ll try and fix it later approach. That it’s monitored properly by whichever authority would have purview of that, whether it’s NICE or the Department of Health or the Chief Medical Officer, or whoever it might be. So that there’s actually accountability around it and proper policies and procedures in place. That’s the key point I would want to see before it was rolled out more widely. P1, FG 1*. *But yes, there was some kind of accreditation process…because the NHS is our service and it looks after us, so we have a lot more faith in it. So if there was some kind of NHS accreditation for private providers, yeah, that would definitely go a long way. And again with GPs as well that would be more inclined to believe what they’re seeing on the bit of paper about your test. P13, FG 3*. *I think I’d need to know how the genetic material is going to be stored, how long they’re going to keep it for and how they’re going to dispose of it? And again, issues around that trust, very specific information about how it’s going to be used and things like that*. Participant 24, FG 5.

### (Mis)understanding towards and awareness of DTC PGx testing

Across the focus groups, participants demonstrated varied levels of understanding of DTC PGx testing, largely related to their prior knowledge of the topic. Participants with no prior experience of PGx generally reported limited awareness of PGx before being invited to attend the focus group, suggesting a broader lack of awareness of PGx amongst the general public. Five participants across two focus groups (FG 4 & 6) had direct experience of privately purchasing PGx tests. These participants had a better understanding and prior knowledge about PGx than those with no experience of PGx or purchasing private health tests. Greater awareness of DTC PGx was not demonstrated across medical specialties.

Participants with no experience of PGx reported being aware of genetic tests conducted within the NHS, or for research purposes, to identify an individual’s risk of diseases such as cancer (e.g. BRCA1/2 gene testing) and dementia etc. However, they were generally unaware that genetic tests could also be used to predict individual responses to medicines or that such tests could be purchased directly by consumers. In FG 2, some participants also demonstrated confusion during the discussion, conflating different types of genetic testing with PGx testing.

Concerns were raised about a potential lack of awareness and support for DTC PGx testing amongst NHS health professionals. In FG 1 & 5, participants suggested that clinicians may require greater education about DTC PGx tests to ensure that test results would be utilised effectively within NHS healthcare. Some participants expressed concerns that paying for DTC PGx testing could represent a waste of money if the results were not utilised because of limited understanding or acceptance among NHS clinicians. Amongst participants who had experience with DTC PGx testing, one participant (P16) shared her experience where an NHS healthcare professional had been dismissive of her PGx test results and declined to use them to guide medication decisions. Participants also reported that some healthcare professionals questioned the accuracy of PGx tests.

Related to the theme of (mis)understanding of PGx testing, the need for greater levels of public awareness of PGx testing was raised organically by participants during FG 1 and 2. Participants emphasised the importance of improved communication and public engagement to foster public awareness. They highlighted the need to clearly explain what PGx testing involves and how it differs from other types of genetic testing. Participants also noted that PGx is a specialist, scientific concept that the general public may be unfamiliar with.

### Altruistic motivations and equity concerns

Overall, participants expressed positive attitudes toward DTC PGx testing. Participants across all focus groups identified both personal and wider societal benefits, including the potential to tailor medicines to individuals to improve patient health outcomes, reduce adverse side effects, minimise medication waste and improve greater resource efficiency. Two participants from different focus groups (FG 2 & 6) used a tailored clothing analogy to convey the perceived benefits of using PGx to personalise medicines. Some participants shared personal experiences where PGx testing had positive impacts for themselves or family members particularly by enabling more individualised prescribing and/or avoiding adverse side effects, leading to better care and health outcomes. Potential benefits of reducing medication waste and cost-saving were identified as important. However, some participants questioned the cost/benefit ratio, raising concerns about whether the cost of PGx testing would outweigh potential cost savings from reduced medication wastage. Altruistic motivations also extended to participants’ views on sharing anonymised PGx data for research purposes, whereby most participants were happy for their anonymised data to be shared on the basis that it would contribute to wider public benefit. However, some participants with no experience of DTC PGx tests or other DTC online tests seemed more cautious about consenting for anonymised data to be shared, due to concerns about trust in private companies (see theme 3 below).

A prominent concern raised by participants across all focus groups were concerns about the potential of DTC PGx to perpetuate inequities in healthcare. As these tests are not currently routinely offered through the NHS, concerns were raised that their cost may render them unaffordable for some individuals, potentially limiting access to those with greater financial means.

### (mis)trust

In relation to the need for greater public awareness and understanding, some participants cautioned that the topic of PGx testing may evoke culturally situated mistrust, related myths, fears and religious beliefs within some communities. To this end, emphasis was placed on being culturally responsive to people’s beliefs to mitigate risks of public mistrust. In FG 2, the discussion indicated that the word ‘genetics’ alone would be enough to evoke fear or distrust, reflecting sensitivities linked to historical experiences of mistreatment.

A number of participants across several focus groups highlighted their personal and broader public mistrust towards private companies – in some cases providing examples of companies they perceived as having questionable ethical practices. Several participants across FG 1, 2 & 3 contrasted this with what they described as greater public trust in the NHS to handle sensitive data securely. Participants with no PGx experience expressed concerns that private companies might sell their genetic data to third parties without their knowledge or permission. Some participants queried what legal protections were in place to prevent this and suggested that stronger regulations may be required. Others highlighted potential risks if insurance companies were able to gain access and use the data in ways that could disadvantage individuals.

Relatedly, the need for regulation of PGx was discussed, in broad terms, across four focus groups (FG1, FG2, FG3 & FG5). Whilst it was not clear how much regulation the participants thought existed, or how much they believed ought to exist, discussions included concerns around the need for regulation of private companies offering DTC PGx testing, the standardisation and accreditation status of laboratories conducting PGx testing, and how genetic data and biological samples are stored and disposed of. Participants emphasised the importance of robust safeguards, national guidelines and regulatory oversight to protect patients. Many felt that such measures would increase both public and professional confidence in PGx testing.

## Discussion

There is currently limited research available on publics’ attitudes towards DTC PGx testing, and little evidence to inform the implementation of DTC PGx testing more widely in the UK. To the authors’ knowledge, this is the first qualitative study to explore the publics’ understanding of and attitudes toward DTC PGx testing in a UK context.

Our findings suggest that awareness and understanding of DTC PGx testing, as well as its governance, remain limited among the general public. Although some participants had prior knowledge of DTC PGx testing, many were confused or conflated different forms of genetic testing during the focus group discussions. This is a notable finding given that our sample tended to be higher educated, with many participants reporting professional backgrounds, including some with clinical experience.

Perhaps the most clinically relevant finding was participants’ perception of the limited knowledge and awareness of PGx testing among healthcare professionals. One participant described an experience in which a healthcare professional declined to accept the results of their DTC PGx. This raised broader concerns amongst participants regarding the acceptance of DTC PGx amongst healthcare professionals and the potential of such tests to be used effectively within the NHS, especially given that there is currently no formal mechanism for DTC PGx data to be incorporated into NHS care pathways. This finding aligns with previous research in this area [14–19]. In the UK, for example, McDermott et al. [16] reported limited awareness among both members of the public and healthcare professionals following a stakeholder workshop that included representatives from NHS England and Genomics England. Similarly, Rafi et al. [18] reported that primary care clinicians expressed concerns about how to educate the workforce and embed PGx into routine clinical practice, highlighting the need for consistent, accessible genomic education for the healthcare workforce, including allied health professionals. In another study, surveyed Flemish pharmacists and physicians demonstrated limited familiarity with both basic theoretical principles and clinical applications of PGx [17]. Moreover, healthcare professionals’ limited confidence in interpreting and applying pharmacogenomic tests and information in clinical practice has been identified as a key barrier to implementation [15, 17, 19]. Our findings are in line with these previous studies, and support recommendations for more education and training for healthcare professionals [20, 21], to help pharmacists in particular to decode PGx tests [22].

Another potential explanation for healthcare professionals’ reluctance to accept DTC PGx results is the concern that patients may independently alter their medication regimens based on misinterpreted PGx information. The traffic-light classification system, in particular, may prompt patients to avoid medication labelled as “red” or to switch to those labelled as “green”, even when their current treatment is effective and well-tolerated [23–25]. Such decisions may be made without considerations of broader clinical factors, which may influence medication effectiveness [22]. Future research could explore stakeholder perspectives on unintended consequences, such as self-directed medication changes, and investigate how these behaviours may impede the integration of DTC PGx results into established clinical care pathways.

Our study also identified concerns regarding data sharing, storage, and the cost of PGx tests. Comparisons with other research on consumer perspectives of PGx indicates that such concerns, particularly those regarding privacy and the sensitivity of genetic information, are widespread [14, 26, 27]. For example, Haddy et al. [14] found that members of the general public raised concerns about the storage and privacy of genetic information, as well as the associated costs, with most participants suggesting that such tests should be funded by the government. Similarly, Edris et al. [26] reported that the majority of citizens surveyed (76.3%) supported partial reimbursement of PGx tests. A lack of a funding mechanism may therefore be a potential barrier to the wider implementation of PGx. In addition, various studies have highlighted that legal, insurance and employment discrimination concerns, if genetic information is disclosed, may further hinder PGx implementation [14, 18, 27–30]. Given the consistency of these findings, our study highlights the importance of providing reassurance to the general public, as well as healthcare professionals, by raising awareness of PGx testing, governance frameworks, safeguards, and regulations that oversee its use – some of which are still under development.

Our findings suggest that the language used when raising awareness of PGx testing and providing reassurance is important. Participants highlighted that certain words or phrases may carry connotations that contribute to misunderstanding and concern if not communicated carefully. Participants emphasised how terminology may be perceived across different cultural backgrounds, as some language associated with genetics may evoke discomfort or reduce engagement with PGx testing. One participant, for example, suggested that the term ‘genetic’ itself could be problematic. Future research is needed to identify language that is both clear and useful for the general public, while remaining sensitive to cultural perspectives and concerns.

Despite these concerns, our study found that most participants expressed positive views toward PGx testing. This aligns with several previous studies. For instance, Edris et al. [26] reported that participants were generally favourable toward PGx testing, even though fewer than one-third had encountered the concept before the survey. Nearly half identified earlier disease treatment and prevention as the main benefit, whereas participants in our study emphasised the value of enabling doctors to personalise medication. Similarly, the potential for PGx testing to support more accurate prescribing and reduce adverse side effects are cited in the literature as benefits and positioned as enablers for implementation [14, 26, 27]. However, Edris et al. [26] also found limited interest in DTC testing, with respondents expressing privacy concerns and preferring testing to be conducted by their healthcare provider rather than commercial companies. This is consistent with our findings regarding mistrust of private companies and a preference for PGx testing to be delivered through the NHS. Likewise, most participants in our study indicated that they would be willing to share their data for PGx research, although views varied regarding who should have access to test results. Similar concerns have been reported elsewhere [27]. Further research is needed to ensure equitable and cost-effective access to PGx testing.

### Limitations

We recruited a diverse sample in terms of age, ethnic backgrounds and health status; however, women were overrepresented, and this may have influenced the results. Furthermore, discussions in the focus groups revealed that many participants were highly educated, and from professional backgrounds. As such, the sample may not be fully representative of the wider British public, specifically those most at risk of health inequities and low health literacy. Consideration of that is important in contextualising these results. Further inclusive research is needed with more marginalised groups.

## Conclusions

This study explored public views of DTC PGx tests among individuals with varying levels of prior PGx experience. The awareness and understanding of PGx were generally low amongst people with no prior experience of DTC PGx testing, with confusion evident around key concepts. However, regardless of their experience, participants expressed generally positive views toward PGx, but support was conditional on clear governance, robust regulation, and transparent data practices being in place. Improving public understanding through accessible communication and development of appropriate quality standards and safeguards will be necessary to ensure equitable and trustworthy implementation of PGx services outside the NHS.

## Materials availability

The datasets used and/or analysed during the current study are available from the corresponding author on reasonable request.

## Supplementary information


Consolidated criteria for reporting qualitative studies (COREQ)


## Data Availability

The datasets used and/or analysed during the current study are available from the corresponding author on reasonable request.

## Footnotes

1 Research for the Future is a NIHR funded online database, which members of the public sign up to, to be alerted about opportunities to get involved in research. https://www.researchforthefuture.org/#
